# Validity of objective methods for measuring sedentary behaviour in older adults: a systematic review

**DOI:** 10.1186/s12966-018-0749-2

**Published:** 2018-11-26

**Authors:** Kristiann C. Heesch, Robert L. Hill, Nicolas Aguilar-Farias, Jannique G. Z. van Uffelen, Toby Pavey

**Affiliations:** 1 Institute of Health and Biomedical Innovation, Queensland University of Technology, Brisbane, Australia; 2 School of Public Health and Social Work, Queensland University of Technology, Brisbane, Australia; 3 Department of Physical Education, Sports and Recreation, Universidad de La Frontera, Temuco, Chile; 4 Department of Movement Sciences, Physical Activity, Sports and Health Research Group, KU Leuven - University of Leuven, Leuven, Belgium; 5 School of Exercise and Nutrition Sciences, Queensland University of Technology, Brisbane, Australia

**Keywords:** Accelerometer, Older adults, Measurement, Sedentary time, Sitting

## Abstract

**Background:**

The evidence showing the ill health effects of prolonged sedentary behaviour (SB) is growing. Most studies of SB in older adults have relied on self-report measures of SB. However, SB is difficult for older adults to recall and objective measures that combine accelerometry with inclinometry are now available for more accurately assessing SB. The aim of this systematic review was to assess the validity and reliability of these accelerometers for the assessment of SB in older adults.

**Methods:**

EMBASE, PubMed and EBSCOhost databases were searched for articles published up to December 13, 2017. Articles were eligible if they: a) described reliability, calibration or validation studies of SB measurement in healthy, community-dwelling individuals, b) were published in English, Portuguese or Spanish, and c) were published or in press as journal articles in peer-reviewed journals.

**Results:**

The review identified 15 studies in 17 papers. Of the included studies, 11 assessed the ActiGraph accelerometer. Of these, three examined reliability only, seven (in eight papers) examined validity only and one (in two papers) examined both. The strongest evidence from the studies reviewed is from studies that assessed the validity of the ActiGraph. These studies indicate that analysis of the data using 60-s epochs and a vertical magnitude cut-point < 200 cpm or using 30- or 60-s epochs with a machine learning algorithm provides the most valid estimates of SB. Non-wear algorithms of 90+ consecutive zeros is also suggested for the ActiGraph.

**Conclusions:**

Few studies have examined the reliability and validity of accelerometers for measuring SB in older adults. Studies to date suggest that the criteria researchers use for classifying an epoch as sedentary instead of as non-wear time (e.g., the non-wear algorithm used) may need to be different for older adults than for younger adults. The required number of hours and days of wear for valid estimates of SB in older adults was not clear from studies to date. More older-adult-specific validation studies of accelerometers are needed, to inform future guidelines on the appropriate criteria to use for analysis of data from different accelerometer brands.

**Trial registration:**

PROSPERO ID# CRD42017080754 registered December 12, 2017.

**Electronic supplementary material:**

The online version of this article (10.1186/s12966-018-0749-2) contains supplementary material, which is available to authorized users.

## Background

Evidence showing the negative health consequences of sedentary behaviour continues to grow. ‘Sedentary behaviour’ (SB) is any awake behaviour done while sitting, reclining, or lying down that requires no more than 1.5 metabolic units of energy expenditure [1]. As well as being associated with psychological distress [2] and poor physical functioning [3], greater amounts of SB have been shown to increase risk of cardiovascular disease incidence and mortality, diabetes incidence, cancer incidence and mortality [4], and all-cause mortality [5–9].

Evidence further suggests a dose-response relationship between subjectively- and objectively-measured SB and poor health outcomes in older adults [3]. Being the most sedentary age cohort [10, 11], older adults are at risk of SB-related diseases. UK researchers found that older adults spent, on average, 11–12 h/day in SB [12]. Half the sampled older adults spent 80% of their time in SB. Similarly, a Canadian study suggested that 94% of older Canadians spent at least 8 h/day sitting [13]. Both these studies measured SB objectively with accelerometers.

An international group of experts in SB research concluded in a consensus statement that future SB research with older adults should provide a better understanding of the correlates of SB to inform intervention studies and that interventions that aim to decrease SB should measure the impact of interventions on SB [14]. Both types of research require accurate measurement of SB, and self-report measures have limited utility for assessing total SB [14]. Indeed, a review of 31 international studies of SB in adults aged ≥60 years found that mean daily SB time was significantly greater when measured with accelerometers (9.4 h/day) than self-report measures (5.3 h/day) [15].

To objectively measure time spent in SB, researchers often use accelerometers [16], which measure changes in acceleration. Although accelerometers were built to measure physical activity, they can indicate low levels of and the absence of movement. However, since movement is determined by acceleration, not body posture [17], they cannot distinguish between sitting and standing still. For this reason, inclinometers (instruments that measure slope or tilt) have been incorporated into some newer accelerometers to detect postures and transitions between postures.

With the addition of inclinometers in accelerometers studies are being conducted to assess the reliability and validity of newer models of accelerometers for assessing SB. Authors of a 2014 systematic review of the use of accelerometers in older adults [18] reported that few accelerometer validations studies had been conducted with older adults [18]. This is an important omission due to the potential to misclassify as non-wear time the large proportions of the day that older adults spend sitting still when standard non-wear algorithms for adults are used [18]. The non-wear algorithm selected for processing data affects estimates of SB [19] because a long string of zeros could represent either (a) time that the monitor was not worn or (b) an extended period in which the monitor wearer is still. The authors of the review [18], therefore, advocated for older adult-specific validation studies. Those authors also reported that to classify SB, accelerometer cut-points ranging from 50 to 500 counts per min (cpm) were being used. The reliability and validity of the cut-points were not discussed. Only one reviewed study included an accelerometer with an inclinometer. The current study systematically reviews the current literature on the reliability and validity of accelerometers with or without inclinometers for measuring SB in older adults.

## Methods

The review was guided by the Preferred Reporting Items for Systematic Reviews and Meta-Analyses (PRISMA) statement [20].

### Search strategy and study selection

The EMBASE, PubMed and three EBSCOhost databases (MEDLINE, CINAHL and SPORTDiscus) were searched for articles published up to December 13, 2017. A three-step process was used. First, KCH searched titles and abstracts using the search terms shown in Table 1. The reference lists of located articles were also searched. Second, two authors (KCH and RLH) independently reviewed the titles and abstracts of each located article to assess its eligibility for inclusion. Disagreements between reviewers were discussed, and consensus reached about which articles would be reviewed in the final step, a review of the full text of articles. For the final step, KCH and RLH independently reviewed the full text of articles and came to consensus about articles to include in the review.

**Table 1:** Search terms

Behaviour (free terms)	Sedentar* Sitting Driving Television TV Screen-time Computer
Measure (free terms)	Acceleromet* ActiGraph ActivPAL GENEActiv Actical Sensewear Actiheart Axivity Inclinom* “Motor sensor” “Activity monitor”
Measurement (free terms)	Valid* Reliab* Sensitivity Specificity Accuracy Precision
Limits	Human English, Spanish, Portuguese Aged 65+

### Inclusion criteria

As in a previous review of measurement in older adults [18], the search was limited to older adults (those aged ≥65 years), although samples with mean ages ≥60 years were included if they were located through the search strategy. Articles were eligible if they: a) described reliability, calibration or validation studies of SB measurement in healthy, community-dwelling individuals, b) were published in English, Portuguese or Spanish, and c) were published or in press as journal articles in peer-reviewed journals. The reliability and validity of accelerometers in populations living in residential care facilities or having a specific disease or disability were not included. Editorials, reviews, and conference abstracts were also not included.

### Reliability and validity of accelerometers

Reliability of accelerometers refers to the consistency in accelerometer readings. Most research on reliability of accelerometers assesses test-retest reliability, which is typically estimated with the intra-class correlation coefficient (ICC) for continuous data [21] including accelerometer data.

Validity refers to the extent to which an accelerometer accurately measures SB. Two types of validity are of interest: criterion and concurrent. Criterion validity refers to the extent to which the findings from the accelerometer agree with the findings produced with a ‘gold standard’ measure [22]. For assessing the criterion validity of an accelerometer, the gold standard is typically calorimetry or direct observation. Concurrent validity refers to the extent to which findings from an accelerometer agree with the findings produced from another type of accelerometer [22]. Because accelerometer counts can be analysed for varying epoch lengths, validity is analysed for specific epochs. Assessing validity requires either (a) using an a-priori cut-point between SB and non-SB behaviours or (b) assessing a range of cut-points. To assess a range of cut-points, researchers typically evaluate which ones optimise sensitivity (the % of epochs classified as SB that were classified by the criterion or concurrent measure as SB) and specificity (the % of epochs classified as not SB that were classified by the criterion or concurrent measure as not SB). The area under the receiver operating characteristics (ROC) curve is often reported as well. Values closer to 1.00 indicate more accurate classification of SB, and values closer to 0.5 indicate less accurate classification of SB [23]. Statistical models (e.g., non-parametric or regression models) or Bland–Altman methods [24] may be used in addition to, or alternatively to, ROC methods, to examine relationships or agreement between the accelerometer of interest and the criterion or concurrent measure. Additional file 1 contains a more detailed discussion of reliability and validity.

### Data extraction

Extracted from eligible articles were: participant and monitor characteristics, study setting, methodological considerations, and results. Extraction tables for the first six studies reviewed were produced independently by two authors (NA, KCH) before then being checked for consistency and accuracy against the original articles by a third author (RLH). For the remaining studies, KCH produced the extraction tables, and RLH checked them for consistency and accuracy against the original articles.

## Results

**Fig. 1:**
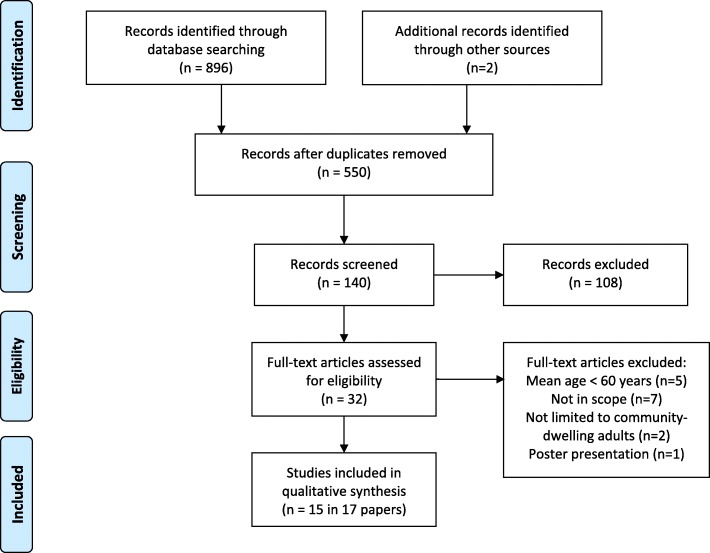
PRISM flow chart

The search identified 550 separate articles (Fig. 1). After articles out of scope were removed, the full text of 32 articles was examined. After applying the selection criteria, 15 different studies of five accelerometer brands (Table 2), reported in 17 papers, were included.

**Table 2:** Description of the accelerometers/inclinometers reported in articles included in the systematic review

Brand and model	Studies in which assessed	Placement of monitor in reviewed studies	Type of monitor	Output available
ActiGraph GT3X+ (ActiGraph LLC, Fort Walton Beach, FL, USA).	[17, 25–31, 33, 34]	Hip, waist, thigh, ankle, wrist	Triaxis accelerometer using piezoresistive and capacitive technology	Activity counts from acceleration signals in vector axis only or in vertical magnitude, a composite measure using the three axes; raw-mode output allows for post-data collection filtering [63]. Filtering and choice of epoch time is done after data collection. Offers a low-frequency extension (LFE) filter, designed to better capture low-intensity activities like sedentary behaviour than the normal filter.
ActiGraph GT3X (ActiGraph LLC, Fort Walton Beach, FL, USA).	[32]	Hip, waist, thigh, ankle, wrist	Triaxis accelerometer using piezoresistive and capacitive technology	Activity counts from acceleration signals in vector axis only or in vertical magnitude, a composite measure using the three axes. Filtering and choice of epoch time must be set before data collection. Offers a low-frequency extension (LFE) filter, designed to better capture low-intensity activities like sedentary behaviour than the normal filter.
ActiGraph 7164 (ActiGraph LLC, Fort Walton Beach, FL, USA).	[35, 36]	Hip	Uniaxis accelerometer using piezoelectric technology	Activity counts that are filtered, digitized and full-wave rectified from acceleration signals in vector axis [63]. Filtering and choice of epoch time must be set before data collection.
Actical (Mini Mitter Respironics, Inc., Bend, OR, USA)	[37]	Waist	‘Omni-directional’ accelerometer using piezoresistive and capacitive technology; most sensitive to motion in one plane	Activity counts that are filtered and digitized from acceleration signals
activPAL (PAL Technologies Ltd., Glasgow, Scotland)	[38]	Thigh	Uniaxis accelerometer using capacitive technology	Classifies activities as sitting/lying, standing or walking
activPAL3 (PAL Technologies Ltd., Glasgow, Scotland)	[38]	Thigh	Triaxis accelerometer using capacitive technology	Classifies activities as sitting/lying, standing or walking
GENEActiv (Activinsights Ltd., Kimbolton, UK)	[39]	Thigh	Triaxis accelerometer with a near-body temperature sensor	Raw-mode data allows for open-source post-data collection filtering
MotionWatch8 (CamNtech, Cambridge, UK)	[40]	Wrist	Triaxis accelerometer using MEMs technology, with ambient light sensor	Activity counts from acceleration signals in a single axis only or in vertical magnitude using epoch-based recoding that uses the three axis; raw-mode data allows for post-data collection filtering

Note: Triaxial accelerometers measure acceleration in vertical axis, antero-posterior and medio-lateral

### Descriptive characteristics of included studies

The ActiGraph accelerometer (ActiGraph LLC, Fort Walton Beach, FL, USA) was examined in 11 studies [17, 25–36] (see Tables 3 and 4). The most commonly-used model of ActiGraph was the GT3X+. Studies included between 20 to 7650 participants, and the mean age of participants ranged from 61 to 78 years. Three studies included only women [27, 28, 30, 33, 34], and the remainder included women and men [17, 25, 26, 29, 31, 32, 35, 36].

**Table 3:** Characteristics and results of studies that examined reliability of ActiGraph models for measuring sedentary behaviour in older adults (mean age ≥ 60 years), ordered from largest to smallest sample size

Study	Participants and data source	Monitor and epochs analysed	Methods	Results for Sedentary Behaviour
Kocherginsky, et al., 2016 [36]	*n* = 2208 57% female (weighted) Mean age = 74.2 y (95% CI 73.7–74.7) USA: Data collected for National Health and Nutrition Examination Survey (NHANES)	ActiGraph 7164 Worn on right hip 60-s epochs	Free-living Activities: VA < 100 cpm Observation period: 7 consecutive days during waking hours Valid hours and days: ≥10 h; ≥1 day Non-wear algorithm: 60 min of consecutive zeros, no allowance for interruptions Analysis: used linear regression to examine variations across days of the week; computed Lin’s concordance coefficients to compare 2-day and then 3-day averages to 7-day average among participants with 7 valid days of data	*Average daily percent of time spent in SB* Monday to Friday: 65.3–65.9% Saturday: 66.3% Sunday: 69.6% Difference between Sunday and Monday to Saturday was significant (*p* < 0.001). Difference between Saturday and Monday to Friday was significant (*p* = 0.045). *Comparison of % time spent in SB between 2 & 3 day averages with 7-day average* Lin’s concordance r: For 2-day vs 7-day: 0.91 For 3-day vs 7-day: 0.94
Keadle et al. (2017) [27]	n: 209 Only females Mean age: 70.6 ± 5.7 y USA: Data collected for an observational ancillary study of participants from the Women’s Health Study, a randomized trial of aspirin and vitamin E to reduce risk of cardiovascular disease and cancer. Data collected after completion of the trial.	ActiGraph GT3X+ Worn on hip 60-s epochs	Free-living Activities: VM < 200 cpm Observation period: two to three 7-day periods over 2–3 y during waking hours Valid hours and days: ≥10 h; ≥4 day Non-wear algorithm: Choi algorithm [61] and ≥ 600 min/day Analysis: computed reproducibility of sitting time across time periods ICCs; used linear mixed models; assessed utility of one 7-day assessment for classifying 2–3 year behaviour by cross-classifying participants using the baseline quartile distribution for SB and the quartile distribution of the average of two follow up assessments	*ICCs (95% CI) over 2–3 years* All participants: 0.75 (0.69, 0.80) Younger: 0.74 (0.66, 0.81) Older: 0.74 (0.65, 0.81) Normal weight: 0.73 (0.65, 0.80) Overweight: 0.76 (0.68, 0.83) Less active: 0.75 (0.67, 0.82) More active: 0.64 (0.54, 0.73) Percent agreement in classification of SB into same quartile at baseline and average of follow-up assessments: 50 and 7% misclassified by ≥2 quartiles
Wanner et al., 2013 [32]	*n* = 65 32 males, 33 females (50.8%) Mean age = 60.8 ± 9.9 y Switzerland: Data collected for ancillary study of the Swiss Cohort Study on Air Pollution and Long and Heart Disease in Adults, after completion of the main study.	ActiGraph GT3X Two worn on right hip 60-s epochs	Free-living Activities: VA < 150 cpm, < 100 cpm and < 200 cpm Observation period: 8 consecutive days Valid hours and days: not reported Non-wear algorithm: 60 min of consecutive zeros, no allowance for interruptions Analysis: compared normal filter to low-frequency extension (LTE) filter using Spearman correlations, Wilcoxon rank sum tests, scatter plots, and Bland–Altman plots; used linear regression to compute correction factors in half the sample and re-analyse results using correction factor	NORMAL VS LTE FILTER FOR VA < 150 CPM *Non-wear time* Spearman r: 0.97 Mean difference: 8.9 ± 13.3; 1.5% ± 2.2%, *p* < 0.001 *Sedentary time (min/day)* Spearman r: 0.96 Mean difference: 25.7 ± 17.6; 4.5% ± 3.1%, p < 0.001 *Other findings* Results for mean differences did not change if cut-point changed to < 100 or < 200 cpm for SB. Plots showed non-wear time and SB time were systematically lower for low-frequency extension vs normal filter. CORRECTION FACTOR FOR LFE FILTER FOR VA < 150 CPM Nonwear min/day: 2.996 + (1.01 x nonwear time from LFE) Sedentary min/day: 62.74 + (0.93 x sedentary time from LFE) COMPARE NORMAL VS LTE USING CORRECTION FACTORS FOR VA < 150 CPM Non-wear time: Mean difference: − 0.8 ± 9.1; − 0.2% ± 1.5, *p* = 0.30 Sedentary time (min/day): Mean difference: 0.1 ± 15.6; − 0.1% ± 2.7%, *p* = 0.72
Hart et al., 2011 [35]	*n* = 52 13 males; 39 females Mean age: 69.3 ± 7.4 y USA: Data collected from participants of larger ongoing study of physical activity patterns.	ActiGraph 7164 Worn on right waist 60-s epochs	Free-living Activities: VA ≤50 cpm Observation period: 21 consecutive days during all waking hours Valid hours and days: not reported Non-wear algorithm: 60 min of consecutive zeros, no allowance for interruptions Analysis: computed reproducibility of sitting time using Spearman-Brown Prophecy Formulas based on ICC; computed RMANOVA to examine differences between days of the week	Number of days of measurement required for: ICC = 0.80: 5 days ICC = 0.85: 7 days ICC = 0.90: 11 days ICC = 0.95: 21 days No significant differences between days of week in time spent in SB (*p* = 0.48)

*Abbreviations*: *cpm* Counts per minute, *IQR* Inter-quartile range, *ICC* Intraclass correlation coefficient; valid hours and days: for free-living studies lasting at least 7 days, number of hours per day and days during observation period that were required for data to be included in analysis; *VA* Vertical axis, *VM* Vector magnitude, *m* Minutes, *s* Seconds, *h* Hours, *y* Years

**Table 4:** Characteristics and results of studies that examined validity and accuracy of ActiGraphs for measuring sedentary behaviour in older community-dwelling, healthy adults (mean age ≥ 60 years), ordered from largest to smallest sample size

Study	Participants	Monitor and epochs analysed	Methods	Results for sedentary behaviour
Keadle et al. (2014) [28]	*n*: 7650 Only females Mean age: 71.4 ± 5.8 y USA: Data collected for an ancillary study of participants from the Women’s Health Study, a randomized trial of aspirin and vitamin E to reduce risk of cardiovascular disease and cancer. Data collected after completion of the trial.	ActiGraph GT3X+ Worn on hip 60-s epochs	Free-living Activities: activities with VA < 100 cpm and VM < 200 cpm Criterion: paper logs Observation period: 7 consecutive days during waking hours Valid hours and days: ≥10 h, ≥1 day and ≥ 4 days Non-wear algorithm: Troiano et al. [62] and Choi et al. [61] Analysis: computed min/day of sedentary activities and used Wilcoxon signed rank sum test to compare output between VA and VM	*VA: min/day (95% CI)* Log+Troiano et al. algorithm: 530.1 (480.1, 578.6) Log+Choi et al. algorithm: 581.6 (521.1, 639.8) *VM: min/day (95% CI)* Log+Troiano et al. algorithm: 474.6 (417.0, 529.6) Log+Choi et al. algorithm: 506.0 (439.2, 570.9) Differences between VA and VM were significant (p < 0.001) Using dates from logs combined with Choi algorithm minimalised missing data and researcher burden. Using algorithm only resulted in misclassification of days when accelerometers were being posted to participants as accelerometer wear days.
Evenson et al. (2015) [33]	*n*: 200 Females only Mean age = 75.5 ± 7.7 y USA: Data collected for a calibration sub-study of participants from the Women’s Health Initiative Long Life Study. Data collected after completion of main study.	ActiGraph GT3X+ Worn on hip 15-s epochs	Laboratory-based Activities: sitting, watching DVD; sitting assembling a puzzle Criterion measure: portable calorimeter Observation period: 7 min per activity Analysis: computed Spearman correlation using normal and low frequency extension for ActiGraph; used ROC analysis to determine optimal cut-points; computed AUC, sensitivity and specificity for normal and low-frequency extension filter	MAXIMISING SUM OF SENSITIVITY+SPECIFICITY *VA: Normal filter* Optimal: 0 counts/15 s AUC: 0.73; Sensitivity: 91%; Specificity: 62% *VA: Low-frequency extension filter* Optimal: 0 counts/15 s AUC: 0.79; Sensitivity: 79%; Specificity: 81% *VM: Normal filter* Optimal: ≤42 counts/15 s AUC: 0.88; Sensitivity: 87%; Specificity: 80% *VM: Low-frequency extension filter* Optimal: ≤65 counts/15 s AUC: 0.90; Sensitivity: 87%; Specificity: 81% BALANCING NUMBER OF FALSE POSITIVES AND FALSE NEGATIVES *VA: Normal filter* Optimal: 0 counts/15 s AUC: 0.73; Sensitivity: 91%; Specificity: 62% *VA: Low-frequency extension filter* Optimal: 0 counts/15 s AUC: 0.79; Sensitivity: 79%; Specificity: 81% *VM: Normal filter* Optimal: ≤12 counts/15 s AUC: 0.88; Sensitivity: 76%; Specificity: 88% *VM: Low-frequency extension filter* Optimal: ≤31 counts/15 s AUC: 0.90; Sensitivity: 71%; Specificity: 88%
Bai et al. (2016) [34]	*n*: 194 Females only Mean age = 75.4 ± 7.7 y USA: Data collected for a calibration sub-study of participants from the Women’s Health Initiative Long Life Study. Data collected after completion of main study.	ActiGraph GT3X+ Worn on hip 1-s epochs	Laboratory-based Activities: sitting, watching DVD; sitting assembling a puzzle Criterion measure: portable calorimeter Observation period: 7 min per activity Analysis: used ROC analysis to compare an activity index created for this study with activity count using the normal filter and LFE, and another method of summarise raw data, the Euclidean Norm Minus One (ENMO); computed AUC also to compare these measures on predicting energy expenditure greater than SB	*Compare watching DVD vs washing dishes or doing laundry, respectively* Activity index: AUC: 0.98, 0.98 Activity count (normal filter): AUC: 0.39, 0.74 Activity count (LTF): AUC: 0.27, 0.87 ENMO: AUC: 0.40, 0.69 *Predicting whether MET is < or ≥ 1.5 MET* Activity index: AUC: 0.96 Activity count (normal filter): AUC: 0.86 Activity count (LFE): AUC: 0.91 ENMO: AUC: 0.85
Chudyk et al., 2017 [26]	*n*: 106 39 men and 76 women Mean age = 74.1 ± 6.4 y Canada: Data collected for Walk the Talk: Transforming the Built Environment to Enhance Mobility in Seniors, a cross-sectional study of older adults living on low incomes. A random stratified design based on neighbourhood walkability was used to recruit older adults who received a provincial government rental subsidy.	ActiGraph GT3X+ Worn on right hip 60-s epochs	Free-living Activities: VA < 100 cpm Criterion: paper log Observation period: 7 days during waking hours Valid hours and days: ≥8 h; ≥4 days Non-wear algorithms: 1. ≥60 min of continuous zeroes; allow for up to 2 min of counts ≤100 counts as non-wear time [62] 2. ≥90 min of consecutive zeroes; allow for up to 2 min of non-zero counts if the interruption was accompanied by 30 consecutive min of 0 counts upstream or downstream [61]. 3. ≥90 min of continuous zeroes; no allow for interruptions, as non-wear time 4. ≥90 min of continuous zeroes; allow for up to 2 min of counts≤50 counts as non-wear time 5. ≥90 min of continuous zeroes, while allowing for up to 2 min of counts ≤100 counts as non-wear time Analysis: used Bland Altman methods to compare logs to non-wear time algorithms	COMPARISON OF EACH ALGORITHM TO LOGS *Mean differences in SB min/day between log and ActiGraph (95% CI)* 1. 37.5 (25.7, 49.3) 2. 5.8 (− 4.4, 16.0) 3. − 4.4 (− 14.6, 5.8) 4. 5.5 (− 4.9, 15.9) 5. 8.1 (− 2.3, 18.5) *95% limits of agreement between log and ActiGraph for wear-time* 1. − 84.6, 159.6 2. − 100.2, 111.8 3. − 110.5, 101.8 4. − 103.0, 114.0 5. − 100.2, 116.4
Koster et al., 2016 [29]	*n*: 62 26 males, 36 females (58.1% females) Mean age = 78.4 ± 5.7 y USA: Data collected for a methodological sub-study of the Developmental Epidemiologic Cohort Study	ActiGraph GT3X+ Worn on hip, right wrist and left wrist concurrently 15-s and 60-s epochs	Free-living Activities: sitting, lying Concurrent measure: ActivPAL worn on right thigh Observation period: 7 full days, with permission given for removal at night Valid hours and days: ≥10 h; ≥1 day Non-wear algorithm: Choi et al. [61] Analysis: for each monitor, used ROC to determine optimal cut-points; computed AUC, sensitivity, specificity, and kappa statistic	STANDARD CUT-OFF POINTS FOR 60-SEC EPOCHS VA < 100 cpm: Sensitivity: 94%; Specificity: 58%; kappa: 0.55 Mean difference: − 114.3 min/day (95%CI -140.5, − 88.1) VM < 200 cpm: Sensitivity: 88%; Specificity: 79%; kappa: 0.68 Mean difference: − 9.9 min/day (95%CI -32.8, 13.0) OPTIMAL 60-SEC EPOCHS *Hip-worn ActiGraph* VA < 22 cpm: AUC: 0.85; Sensitivity: 85%; Specificity: 74%; kappa: 0.59 Mean difference: − 5.0 min/day (95%CI -29.5, 19.5) VM < 174 cpm: AUC: 0.89; Sensitivity: 87%; Specificity: 80%; kappa: 0.67 Mean difference: 2.5 min/day (95%CI -20.4, 25.5) *Wrist-worn ActiGraph* VM < 2303 cpm (dominant wrist): AUC: 0.86; Sensitivity: 81%; Specificity: 78%; kappa: 0.58 Mean difference: 30.2 min/day (95% CI 10.7, 49.6) VM < 1853 cpm (non-dominant wrist): AUC: 0.86; Sensitivity: 82%; Specificity: 77%; kappa: 0.57 Mean difference: 22.6 min/day (95%CI 0.5, 44.6) OPTIMAL 15-S EPOCHS *Hip-worn ActiGraph* VA < 1 count/15 s: AUC: 0.75; Sensitivity: 87%; Specificity: 61%; kappa: 0.50 Mean difference: − 53.4 min/day (95%CI -76.4, − 30.4) VM < 20 counts/15 s: AUC: 0.83; Sensitivity: 83%; Specificity: 73%; kappa: 0.56 Mean difference: 12.8 min/day (95%CI -8.8, 34.4) *Wrist-worn ActiGraph* VM < 517 counts/15 s (dominant wrist): AUC: 0.81; Sensitivity: 75%; Specificity: 75%; kappa: 0.48 Mean difference: 64.7 min/day (95%CI 45.7, 83.7) VM < 376 counts/15 s (non-dominant wrist): AUC: 0.81; Sensitivity: 75%; Specificity: 74%; kappa: 0.47 Mean difference: 64.7 min/day (95%CI 44.3, 85.0)
Rosenberg et al. (2017) [30]	*n*: 39 Only females Mean age = 69.4 (range: 56–94) USA: A convenience sample	ActiGraph GT3X+ Worn on right hip 60-s epochs	Free-living Activities: sitting and riding in a vehicle Criterion: DO (images produced via SenseCam camera worn on lanyard around neck) Observation period: 7 days during waking hours Valid hours and days: not reported Non-wear algorithms: Choi et al. [61] Analysis: used machine learning algorithm to classify activities (sitting and riding in vehicle analysed separately), computed sensitivity and specificity	*Sitting* Sensitivity: 89%; Specificity: 91% Median counts = 0 (IQR: 0, 17), indicating that sitting occurred at a lower intensity than would be detected by existing threshold of < 100 cpm *Riding in a vehicle* Sensitivity: 84%; Specificity: 99% Median counts = 72 (IQR: 21, 177), indicating that riding occurred at a higher intensity than would be detected by threshold of < 100 cpm
Aguilar-Farias et al. (2014) [17]	*n*: 37 13 males, 24 females (64.9% female) Mean age: 73.5 ± 7.3 y Australia: A convenience sample recruited mainly via flyers displayed at senior centres and exercise centres, and emails to university staff	ActiGraph GT3X+ Worn on right hip 1-s, 15-s, 60-s epochs	Free-living Activities: sedentary activities defined as VM and VT counts being below cut-points set separately for 1-s, 15-s and 1-m epochs as follows: 1-s (< 1 to < 10 in increments of 1 counts/s), 15-s (< 1 to < 100 in increments of 5 counts/15 s) and 60-s epochs (< 1 to < 400 in increments of 25 cpm) Concurrent measure: ActivPAL3™ worn on right thigh Observation period: 7 consecutive days during waking hours Valid hours and days: ≥10 h; ≥5 days Non-wear algorithms: 90-min of consecutive zeros with no interruptions allowed Analysis: used ROC to determine optimal cut-points, calculated AUC and computed sensitivity, specificity, percent correctly classified, mean bias (min/day)	OPTIMAL CUT-POINTS FOR VA *< 1 count/s* AUC:0.67; Sensitivity:92%; Specificity:43% Correctly classified: 74% Mean bias:156.61 (95%LoA: − 34.5, 347.7) *< 10 counts/15 s* AUC:0.70; Sensitivity:84%; Specificity:65% Correctly classified: 79% Mean bias: − 4.29 (95%LoA: − 141.3, 132.8) *< 25 cpm* AUC:0.79; Sensitivity:83%; Specificity:75% Correctly classified: 80% Mean bias:4.81 (95%LoA: − 157.2, 166.8) OPTIMAL CUT-POINTS FOR VM *< 1 count/s* AUC:0.73; Sensitivity:85%; Specificity:62% Correctly classified: 76% Mean bias: 0.98 (95%LoA: − 113.4, 15.4) *< 70 counts/15 s* AUC:0.79; Sensitivity:87%; Specificity:70% Correctly classified: 82% Mean bias:0.80 (95%LoA: − 188.5, 120.1) *< 200 cpm* AUC:0.84; Sensitivity:89%; Specificity:79% Correctly classified: 85% Mean bias:18.05 (95%LoA: − 107.2, 143.3)
Sasaki, 2016 [31]	*n* = 35 14 males; 21 females Mean age: 70.8 ± 4.9 y USA: A convenience sample	ActiGraph GT3X+ Worn on dominant hip, wrist and ankle 20-s epochs	LABORATORY-BASED Activities and observation period: performed sitting and lying down postures (30 s each); sat doing crossword puzzles or playing cards (5 min) Criterion measure: DO FREE-LIVING (*N* = 15) Activity: SB Criterion measure: DO (trained observers coded activities with continuous focal sampling software in a personal digital assistant) Observation period: 2–3 h Analysis: output used to train random forest (RF) and support vector machine (SVM) algorithms to classify activities; different algorithms developed for different body placement of monitor; computed percent correct classification	% CORRECT CLASSIFICATION AS SB *Lab-based algorithms applied to lab and free-living conditions: using 20-s epochs* SVM hip: Lab: 92%; Free: 68% SVM wrist: Lab: 97%; Free: 73% SVM ankle: Lab: 92%; Free: 79% RF hip: Lab: 92%; Free: 62% RF wrist: Lab: 93%; Free: 71% RF ankle: Lab: 89%; Free: 76% *Free-living-based algorithms applied to free-living conditions: using 20-s epochs* SVM hip: Free: 82% SVM wrist: Free: 75% SVM ankle: Free: 87% RF hip: Free: 81% RF wrist: Free: 81% RF ankle: Free: 84% *Free-living-based algorithms applied to free-living conditions: using other epochs* 5-s epochs: hip: 79%; wrist: 70%; ankle: 78% 10-s epochs: hip: 82%; wrist: 74%; ankle: 82% 15-s epochs: hip: 82%; wrist: 75%; ankle: 87% 30-s epochs: hip: 82%; wrist: 78%; ankle: 87% RF ALGORITHMS 5-s epochs: hip: 72%; wrist:73%; ankle: 75% 10-s epochs: hip: 77%; wrist: 77%; ankle: 81% 15-s epochs: hip: 81%; wrist: 81%; ankle: 84% 30-s epochs: hip: 83%; wrist: 84%; ankle: 86% FREE-LIVING FOR HIGHEST OVERALL CLASSIFICATION RATES ACROSS ANKLE, HIP and WRIST ALGORITHMS, AT 30-S EPOCHS Sensitivity and specificity: Ankle (SVM): 82%; 94% Hip (RF): 79%; 93% Wrist (RF): 69%; 92%
Bourke et al., 2016 [25]	*n*: 20% females not provided Mean age = 76.4 ± 5.6 y Norway: A convenience sample	ActiGraph GT3X+ Worn on right hip 5-s epochs	LABORATORY-BASED Activities: semi-structured protocol that included sitting and lying Criterion measure: DO (video camera) Observation period: in one session FREE-LIVING Activities: sitting and lying as part of tasks requested by researchers + normal routine Criterion measure: DO (camera on head) Observation period: partial day Analysis: used laboratory-based and free-living data together to assess % correctly classified using researcher-developed algorithm	Across both conditions % correct classification: Sitting: 75% Lying: 51%

*Abbreviations*: *AUC* Area under the ROC curve that is used to evaluate classification accuracy, *cpm* Counts per minute, *IQR* Inter-quartile range, *ICC* Intraclass correlation coefficient, *LoA* Low-frequency extension filter, *LTE* Limit of Agreement, *PPV* Positive predictive value, *ROC* Receiver operator characteristic analysis; valid hours and days: for free-living studies lasting at least 7 days, number of hours per day and days during observation period that were required for data to be included in analysis;*VA* Vertical axis, *VM* Vector magnitude, *m* Minutes, *s* Seconds, *h* Hours, *y* Years

One study each assessed the Actical, the ActivPAL3, the GENEActiv, and the MotionWatch 8. These studies included men and women. The Actical study [37], the largest of the four, included 200 participants with a mean age of 64 years. The ActivPAL3 study [38] included 53 participants with a mean age of 75 years. The GENEActiv study [39] included 40 participants with a mean age of 74 years. Last, the MotionWatch8 study [40] included 23 adults with a mean age of 70.

### Reliability of the ActiGraph

Test-retest reliability of the ActiGraph in free-living conditions was assessed in three studies (Table 3). In each study, an ActiGraph was worn on the hip, and the data were analysed in 60-s epochs. In a 21-day study of the reliability of the ActiGraph 7164, Hart et al. [35] found that 5 days of measurement was required to attain an acceptable level of reliability (ICC = 0.80) in measuring SB in older adults. SB was defined as vertical axis (VA) ≤50 cpm. In a 7-day study of the ActiGraph 7164 [36], 2- to 3-day protocols provided reliable estimates of the percentage of time spent in SB, but the authors concluded that estimates should be adjusted for greater time spent in SB on weekend days than on weekdays. SB was defined as VA < 100 cpm. In a study of the 2–3 year test-retest reliability of the ActiGraph GT3X+ [27] with SB defined as vertical magnitude (VM) < 200 cpm, reliability was slightly lower for daily minutes spent in SB than seen in the other studies, but excellent given the long intervals between measurement periods (ICC = 0.75). Overall, these findings provide uncertainly about the number of days required for reliable estimates of SB. The two studies that directly addressed the required number of days were both conducted with the ActiGraph 7164, and results could be different in newer models. Also, differences in data collection periods, cut-points used to determine SB, non-wear time algorithms, and axis used to assess SB (one or three) make direct comparison among studies problematic. Therefore, the evidence to date does not provide a clear indication of the number of days of measurement with an ActiGraph that is required for older adults.

A fourth study [32] assessed the reliability of two filters that can be used with the ActiGraph GT3X: the normal filter, which is the standard filter, and the low-frequency extension (LTE) filter, which was designed to better capture low-intensity activities. Participants wore two monitors on a hip for 8 days in free-living conditions. For analysis 60-s epochs were used. The researchers found large mean differences between filters in min/day and % of time in SB with estimates systematically lower when the LFE filter was used than when the normal filter was used. The results were the same when the VA cut-point for SB was changed from < 150 cpm to < 100 cpm or < 200 cpm. The results indicate that the estimates of time spent in SB differ depending on the filter selected, and therefore, results of studies that use one type of filter are not comparable to studies that use the other type.

### Validation and accuracy of ActiGraph cut-points for classifying SB

All validation studies of the ActiGraph assessed the GT3X+. With the ActivPAL as the concurrent measure, two 7-day studies showed moderate to good accuracy of the hip-worn ActiGraph for classifying SB in free-living conditions [17, 29] (Table 4). Aguilar-Farias et al. [17] reported that the optimal cut-points for the VA were < 1 count/s, < 10 counts/15 s, and < 25 cpm. The percentage of correctly classified SB epochs was good (74–80%). For VM, the optimal cut-points were < 1 count/s, < 70 counts/15 s, and < 200 cpm. For VA and VM, accuracy was better for the cpm threshold than for the 1-s and 15-s thresholds. Koster et al. [29] also showed better accuracy for 60-s epochs than 15-s epochs: for a 60-s epoch a VA cut-point of < 22 cpm and a VM cut-point of < 174 cpm were optimal. These values are slightly lower than those reported by Aguilar-Farias et al. [17]. The researchers noted that if the commonly-used VA cut-point of < 100 cpm had been used, there would have been an overestimation of SB of 114 min/day. However, the commonly-used VM cut-point of < 200 cpm produced an overestimate of only 10 mins/day of SB.

Koster et al. [29] also computed optimal SB cut-points for the wrist-worn GT3X+, and these showed comparable accuracy properties to those reported when the monitor was worn on the hip. The most accurate VM cut-points were < 2303 cpm on the dominant wrist and < 1853 on the non-dominant wrist. Their optimal 60-s epoch cut-points for hip- and wrist-worn monitors produced more accurate results than the use of their optimal 15-s epoch cut-points.

Using data collected in a laboratory setting, Evenson et al. [33] computed optimal SB cut-points for the GT3X+. The criterion measure was portable calorimeter. With 15-s epochs, VM was more accurate than VA for classifying SB, and the LFE filter was not substantially better than the normal filter. Accuracy was highest when the sum of sensitivity and specificity with either a normal filter (optimal cut-point: ≤42 counts/15 s) or LFE filter (optimal cut-point: ≤65 counts/15 s) was maximized. For another analysis of that study’s data, Bai et al. [34] showed that activity counts with the normal or LFE filter generally performed poorly against portable calorimeter in differentiating between SB and two light activities.

These findings suggest that for using ActiGraph GT3X+ worn at the hip, 60-s epochs and VM provide more accurate estimates of SB than shorter epochs or VA, respectively. The findings from the studies in free-living conditions suggest that VM < 200 cpm provides valid estimates of SB time. However, if VA is used, the cut-point should be much smaller than typically used (e.g., < 22–25 cpm). One study also provided VM cut-points for ActiGraph GT3X+ worn on the wrist. Moreover, findings suggest that the use of LFE does not substantially improve the estimates of SB time produced with a normal filter although caution is warranted in extrapolating the laboratory-based findings to free-living conditions.

### Validity and accuracy of machine learning algorithms for classifying SB with the ActiGraph

Three studies assessed whether machine learning algorithms accurately classify SB (Table 4). In a study by Rosenberg et al. [30] participants wore a camera on a lanyard around their necks while wearing the ActiGraph on a hip for 7 days in free-living conditions. Epochs were set to 60 s. The researchers showed that a machine learning algorithm using ActiGraph output could more accurately differentiate SB from non-sedentary behaviours than other methods. The researchers also reported that the median counts for sitting were much lower than would be detected by a < 100 cpm threshold and the median counts for riding in a vehicle were higher than would be detected at that threshold. This finding further supported the superiority of the algorithm over the use of a set cut-point.

Sasaki et al. [31] compared two machine learning algorithms for classifying activities and examined whether algorithms created in laboratory conditions were as accurate as those created in free-living conditions for detecting SB in free-living conditions. Direct observation was the criterion measure for both conditions. Using 20-s epochs for ActiGraphs worn on the hip, wrists and ankle, the laboratory-based algorithms were not as accurate as ones developed in free-living conditions (over 2–3 h, % of minutes correctly classified as SB was > 80% except for one wrist-worn algorithm). For algorithms produced under free-living conditions, the researchers showed that the accuracy in correctly classifying minutes as SB was optimal (defined as 80% of minutes correctly classified as SB) when the ActiGraph was placed at the hip or ankle (not wrist) and 15- or 30-s epochs were used. The highest overall classification rates were for 30-s epochs.

These machine-learning algorithms performed substantially better than an algorithm developed for another study [25]. As in the study by Sasaki et al. [31], direct observation was the criterion measure for both laboratory-based and free-living conditions, which were conducted in sessions lasting less than 1 day. Epochs were set to 5 s, which the findings by Sasaki [31] suggest is not as accurate as using longer epoch lengths. Also, data from laboratory-based and free-living components of the study were combined for analysis, which could have negatively impacted the findings, given that Sasaki [31] found differences in accuracy between laboratory-based versus free-living algorithms.

Overall, these findings indicate that machine learning algorithms may provide more accurate estimates than cut-points, particularly when these algorithms use large epochs, 30-s and 60-s, with data from free-living conditions.

### Validity and reliability of other brands of accelerometers

Three other monitors underwent testing in laboratories: the activPAL3, GENEActiv, and MotionWatch 8 (Table 5). Klenk et al. [38] compared the newer activPAL3 to the original activPAL. Both were worn on the thigh. The researchers reported high agreement (98%) between monitors, but for a 24-h period, the researchers calculated, between monitors, a mean difference of 45 min in time spent sitting/lying. The findings suggest that the two monitors should not be used interchangeably for assessing SB. In the second study, Wullems et al. [39] validated the GENEActiv, worn on the thigh, against indirect calorimetry. Three cut-point algorithms and one machine learning algorithm performed well at classifying SB. In the final study, Landry [40] conducted the first validation of the MotionWatch 8. In that study, participants wore two watches on non-dominant wrists while performing SB and other activities. The watch was validated against portable calorimeter. The optimal cut-point for SB was ≤179 cpm.

**Table 5:** Characteristics and results of studies that examined validity and accuracy of other accelerometers and inclinometers for measuring sedentary behaviour in older community-dwelling, healthy adults (mean age ≥ 60 years), ordered from largest to smallest sample size

Study	Participants	Monitor and epochs analysed	Methods	Results for Sedentary Behaviour
Hutto, et al., 2013 [37]	*n*: 200 85 males, 115 females (58% female) Mean age = 63.5 ± 8.3 USA: Data collected as subs-tudy of The Reasons for Geographic and Racial Differences in Stroke Study, a national cohort study of racial and regional disparities in stroke risk and mortality	Actical Worn on waist 60-s epochs	Free-living Activities: VA ≤100 cpm Concurrent measure: wear-time logs Observation period: 7 consecutive days during waking hours Valid hours and days: ≥10 h; ≥4 days Non-wear algorithms: 60-, 90-, 120-, 150-, and 180-min of consecutive zeros, no allowance for interruptions Analysis: compared algorithms for estimating wear and non-wear time by computing min/day and % of total wear time in SB for each algorithm	ALGORITHMS USING THE FOLLOWING MINUTES OF CONSECUTIVE ZEROES TO MEASURE NON-WEAR TIME *Min/day were classified as SB* 60-min: 618 ± 81 90-min: 649 ± 88 120-min: 667 ± 97 150-min: 675 ± 98 180-min: 679 ± 101 *% of total wear time was SB* 60-min: 75 ± 10 90-min: 77 ± 10 120-min: 77 ± 10 150-min: 77 ± 10 180-min: 77 ± 10
Klenk et al. (2016) [38]	*n*: 53 31 males and 22 females (41.5% female) Mean age: 75.3 ± 4.6 y Germany: Data collected as sub-study of ActiFE-Ulm study, a national cohort study of physical activity and health outcomes	ActivPAL3 Worn on left thigh	Laboratory-based Activities: 2 bouts of sitting; 2 bouts of lying Concurrent measure: ActivPAL worn on left thigh concurrently Observation period: 10 s per bout of activity with a total observation period of mean 156.5 min ± 16.5 Analysis: computed agreement between the 2 monitors by (1) using Bland Altman methods and (2) computing for the activPAL sitting/lying category the degree to which activPAL3™ identified sitting/lying or different activities	Mean difference: − 2.00 s (±2 SD: 3.52) Median agreement: 98.0% (IQR 95.9–99.0) Expected difference in SB duration (95% CI) for 24-h measurement: − 44.5 min (− 69.9, − 20.0)
Wullems et al. (2017) [39]	*n*: 40 20 males, 20 females (50.0% female) Mean age = 73.5 ± 6.3 y UK: Convenience sample	2 GENEActiv Worn on thigh	Laboratory-based Activities: sitting in a chair; lying down Observation period: 4 min per activity Criterion: indirect calorimeter Analysis: compared machine learning algorithm to three cut-point algorithms for classifying intensities of activities (if MET value ≤1.5 and position was not upright), computed sensitivity and specificity	*3 methods using cut-point algorithms* Sensitivity: 99.3–99.9% Specificity: 99.7% Accuracy: 99.5–99.8% *Random Forest machine learning* Sensitivity: 99.9% Specificity: 99.2% Accuracy: 99.6% Other findings: Participant-specific accuracies resulted in perfect score (100%) for all algorithms for SB.
Landry et al., 2015 [40]	*n*: 23 7 males and 16 females (69.6% female) Mean age: 70.0 ± 6.6 y Canada: Convenience sample recruited through newspaper advertisements, brochures distributed at community centres, and word of mouth	MotionWatch 8 Two worn on non-dominant wrist 60-s epochs	Laboratory-based Activities: sitting in a chair; lying down Criterion: portable calorimeter Observation period: 5-min per activity Analysis: used ROC to determine optimal cut-points with SB defined as < 1.5 MET, computed AUC, sensitivity, specificity, PPV, negative predicted value	AUC: 0.81 (95%CI: 0.78, 0.85) Optimal cut-point for SB: ≤178.5 (*d*^*2*^ = 0.14) Sensitivity: 78% Specificity: 70% Accuracy: 70% PPV: 30% Negative predictive value: 94%

*Abbreviations*: *AUC* Area under the ROC curve that is used to evaluate classification accuracy, *cpm* Counts per minute, *IQR* Inter-quartile range, *ICC* Intraclass correlation coefficient, *LoA* Low-frequency extension filter, *LTE* Limit of Agreement, *PPV* Positive predictive value, *ROC* Receiver operator characteristic analysis, *SD* Standard deviation; valid hours and days: for free-living studies lasting at least 7 days, number of hours per day and days during observation period that were required for data to be included in analysis; *VA* Vertical axis, *VM* Vector magnitude, *m* Minutes, *s* Seconds, *h* Hours, *y* Years

These early validity assessments indicate that the newest models of non-ActiGraph monitor brands show promise for classifying SB in older adults. Future studies in free-living conditions are needed to verify whether these findings hold in real-life conditions.

### Accuracy of non-wear time algorithms for classifying SB

Two studies [26, 28] examined the influence of the non-wear-time algorithm selected on the classification of SB (Table 4). Both studies used the ActiGraph GT3X+, worn on the hip during free-living conditions, for 7 days. Both studies used 60-s epochs and defined SB as VA < 100 cpm with one study [28] also requiring VM < 200 cpm. For determining non-wear time Keadle et al. [28] found that the use of a paper log with the Choi algorithm [41] was better than using this algorithm only or using another algorithm with our without a log, for minimising missing data. The algorithms examined used ≥60 min threshold. Dates on logs were needed because accelerometers were mailed to participants. Without a log of wear-time dates, the algorithm misclassified ‘wear’ days as ‘in the mail’ days. Chudyk et al. [26] showed that algorithms that counted ≥90 min of consecutive zeroes as non-wear time were more accurate in estimating SB compared with ones using a ≥ 60 min threshold.

In contrast, Hutto et al. [37] examined the accuracy of the wrist-worn Actical in producing estimates of SB across non-wear estimation algorithms (see Table 5). Participants worn the monitor for 7 days and kept wear-time logs. SB was defined as VA ≤100 cpm. The analysis showed that estimates of time spent in SB were similar among algorithms that counted ≥120 min of consecutive zeros as non-wear time (with no allowance for intervals of non-zero cpm). Using 60- or 90-min intervals produced underestimations of time in SB.

In summary, for the ActiGraph, it is not clear whether treating ≥60 min or ≥ 90 min of consecutive zeroes as missing data is best for accurately classifying SB, but findings from the only study that compared the two indicated that the ≥90 min is optimal. For the Actical, initial findings indicate that non-wear time should include a longer string of consecutive zeros (e.g., ≥120 min of consecutive zeros).

## Discussion

Accurate measurement of SB is critical for the evaluation of patterning and prevalence of this behaviour and of future health promotion strategies aimed at decreasing SB. The aim of this systematic review was to assess the validity and reliability of accelerometers for the assessment of SB in older adults. Fifteen eligible studies were identified.

Comparison among studies of older adults in this field is challenging due to the heterogeneous assumptions used for the measurement parameters. For example, studies varied greatly in what constituted a valid day, number of measurement days, epoch length, use of VA or VM, and cut-point thresholds. Validity was assessed predominately using the ActiGraph GT3X+. Reliability was assessed using the ActiGraph GT3X+ and 7164. Most studies used accelerometers worn on the hip and utilized 60-s epochs.

Test-retest reliability estimates of the ActiGraph 7146 were similar to estimates found in younger adult populations (ICC 0.74–0.94) [42]. However, the number of days required for a reliable estimate of SB in older adults remains uncertain. Although 2–5 days are suggested from the studies reviewed, these estimates are drawn from only two studies, which used an older model accelerometer (7164); therefore, estimates may be less relevant for newer models. More research is required with newer model accelerometers, to determine a reliable number of wear days in older adults. Decisions about the number of wear-days selected for use in this population must also consider that adherence to the generally recommended 7-day wear-time protocols can be burdensome to older adults [36]. A move to a wrist location, which would avoid the need for removal when changing clothes, showering or sleeping, may result in greater compliance with wear-time requirements [43]. Data from NHANES shows compliance with waist-worn protocols of 40–70% but 70–80% for wrist-worn protocols [44]. However, further investigation into the validity and reliability of wrist-worn accelerometers in older adults is required before their use in research with this population is recommended.

Another consideration is the selected cut-points, which can greatly impact the amount of SB recorded. For example, Gorman and colleagues [18] reported that in a population of older women the mins/day spent in SB ranged from 475 when the cut-point for SB was ≤50 cpm to 665 when the cut-point was < 500 cpm. The current review found only two studies that examined appropriate ActiGraph SB cut-points for older adults in free-living conditions. The evidence from these studies of the GT3X+ suggest a cut-point of < 200 cpm with VM and a cut-point of < 22–25 cpm with VA, when the wear location is the hip and the normal filter is used. However, a commonly-accepted cut-point for adults is VA < 100 cpm. Results of a study that analysed GT3X data from office workers (mean age = 47 years) indicated that a cut-point of < 150 cpm was optimal although < 100 cpm was acceptable [45]. The analysis used VA and a normal filter. The results of more recent studies in younger adults (university employees and university students) that used LFE with the GT3X+ suggested that a < 65 cpm cut-point with VA [46] or a < 150 cpm cut-point with VM [47] were appropriate. In short, the totality of evidence provides early indications that higher cut-points are needed for assessing SB in older adults than in their younger counterparts. Differences between age groups in these cut-points could indicate that estimates of movement patterns using cut-points may vary for different life stages, due to dissimilar balance and gait speed as well as the nature and contexts of movement [48, 49].

Other factors influencing estimated SB include decisions about epoch length and non-wear-time algorithms. From the findings of this review, it appears that 60-s epochs are the most accurate to use with older adults for assessing SB with the ActiGraph are, and a 90+ minute non-wear time algorithm may be most accurate although this result is from only one study. In younger adults 60-s epochs and 60+ minutes of non-wear time [50] are generally used in analysis. Others [18] have suggested that 60+ non-wear time algorithms are not likely to be appropriate for older adults because the large percentage of the day that older adults spend sitting quietly could be misclassified as non-wear time. Although cut-points remain the most common method for accelerometer data reduction [18], the choice of cut-points and their inherent assumptions (e.g., epoch length, non-wear time) greatly impact validity and reliability. Assumptions also affect comparisons of SB and PA estimates in other life stages (e.g., children [51] and adults [52]).

A developing alternative approach for estimating SB is the use of machine learning, or pattern recognition [53]. Three studies in this review indicated that machine learning algorithms provide more accurate estimates of SB than other methods when using a 30-s or 60-s epoch. The findings from these studies further suggest that using ActiGraph data from free-living conditions are more accurate than laboratory data in classifying activity as SB with machine learning. These findings support those from similar studies in younger adults [54]. As highlighted by Sasaki and colleagues [31], there is a need for more rigorous field-based assessment of SB using machine learning as few such assessments have been conducted in older or younger adults.

Of the non-ActiGraph monitors examined, early validity assessments of the ActivPAL3, GENEActiv and MotionWatch 8 in older adults show promise for classifying SB in older adults. However, studies were conducted in laboratories; studies with older adults in free-living conditions are needed to verify whether findings hold in real-life conditions. In contrast, some studies of the GENEActiv [43, 55, 56] and the ActivPAL or ActivPAL3 [45, 57–60] in younger samples have been conducted in free-living conditions. These suggests that the wrist-worn GENEActiv and thigh-worn ActivPAL/ActivPAL3 monitors may be suitable for estimating population-levels of SB, at least in younger adults, and therefore, the suitability of these for use in older adults merits further exploration. Also noteworthy from this review are the inter-brand differences for older adults, with findings suggesting that non-wear time is best captured for the Actical using the rule of ≥120 min of consecutive zeros, compared with ≥90 min for the ActiGraph. Standard algorithms for analysis of ActiGraph data call for ≥60 min [61, 62].

### Strengths and limitations

This review used a systematic search of multiple bibliographic databases. The major strength is the inclusion of studies of all brands and models of accelerometers that examined the reliability or validity of accelerometers. Previous reviews have tended to narrow the focus to one monitor brand, ActiGraphs. This is reasonable given that most validation studies have been done with ActiGraphs [18]. However, for a comprehensive review of the reliability and validity of all accelerometers that are being used with older adults, it is important to include all accelerometers. Another strength of this review was the inclusion of papers published in Spanish and Portuguese in addition to those published in English. However, all studies that met the inclusion criteria were published in English.

Two limitations of the review should be noted. First, studies were not rated on their quality. Although there are reporting lists for diagnostic studies, we are not aware of quality rating lists for studies into measurement characteristic that are relevant across different models and brands of monitors or monitors using different assumptions. However, one strength of the included studies was the rigorous designs used overall, with most validation studies reporting their assumptions, collecting data in free-living conditions across multiple days, and using appropriate concurrent or criterion measures. Most studies also described criteria for inclusion of data in analysis and use of non-wear time algorithms. Second, we only included studies of healthy, community-dwelling older adults. Although the literature on the validity of accelerometers in other older populations (e.g., residential care facilities or with specific diseases) is growing, the assumptions underlying analysis is likely to be differ under those situations.

## Conclusions

This paper reviewed the literature on the reliability and validity of accelerometers for measuring SB in older adults. The number of studies identified was small, 15 studies in 17 papers. Most studies assessed hip- or waist-worn ActiGraphs. The studies of validity assessed the GT3X+ model. These studies indicated that analysis of 60-s epochs and a VM cut-point of < 200 cpm in free-living conditions or the use of 30-s or 60-s epochs with machine learning algorithms provide the most valid SB estimates. Non-wear algorithms of 90+ consecutive zeros were suggested. This finding indicates that the criteria researchers use for classifying an epoch as sedentary instead of as non-wear time (e.g., the non-wear algorithm used) may need to be different for older adults than for younger adults. However, this conclusion is based on the findings of only one study. Two studies of an older model ActiGraph (7164) examined the number of wear-days that would be required for an acceptable reliability estimate (> 0.80). Results varied (2–5 days), and the relevance of these findings to new models is unknown. Also noteworthy was the paucity of studies on the reliability and validity of other accelerometer brands. Overall, more older-adult-specific validation studies of accelerometers are needed, to inform future guidelines on the appropriate criteria to use for analysis of data from various accelerometer brands.

## Additional file

Additional file 1: Definitions and descriptions of test-retest reliability and validity for assessment of accelerometers and inclinometers. (DOCX 15 kb)

## Abbreviations

cpm: Counts per minute
min: Minute
s: Second
SB: Sedentary behaviour
VA: Vertical axis
VM: Vertical magnitude

## References

[1] Tremblay MS, Aubert S, Barnes JD, Saunders TJ, Carson V, Latimer-Cheung AE, Chastin SFM, Altenburg TM, Chinapaw MJM (2017). Sedentary behavior research network (SBRN) – terminology consensus project process and outcome. Int J Behav Nutr Phys Act.

[2] Hamer Mark, Coombs Ngaire, Stamatakis Emmanuel (2014). Associations between objectively assessed and self-reported sedentary time with mental health in adults: an analysis of data from the Health Survey for England. BMJ Open.

[3] Copeland JL, Ashe MC, Biddle SJH, Brown WJ, Buman MP, Chastin S (2017). Sedentary time in older men and women: a critical review of measurement, associations with health, and interventions. Br J Sports Med.

[4] Biswas A, Oh PI, Faulkner GE, Bajaj RR, Silver MA, Mitchell MS, Alter DA (2015). Sedentary time and its association with risk for disease incidence, mortality, and hospitalization in adults: a systematic review and meta-analysis. Ann Intern Med.

[5] Biswas A, Alter DA (2015). Sedentary time and risk for mortality. Ann Intern Med.

[6] Grunseit AC, Chau JY, Rangul V, Turid Lingaas H, Bauman A. Patterns of sitting and mortality in the Nord-Trondelag Health Study (HUNT). Int J Behav Nutr Phys Act. 2017;14:8. 10.1186/s12966-016-0457-8 PMC5267382 28122625

[7] Matthews CE, Moore SC, George SM, Sampson J, Bowles HR (2012). Improving self-reports of active and sedentary behaviors in large epidemiologic studies. Exerc Sport Sci Rev.

[8] Pavey TG, Peeters GG, Brown WJ (2015). Sitting-time and 9-year all-cause mortality in older women. Br J Sports Med.

[9] de Rezende LFM, Rey-López JP, Matsudo VKR, Luiz OC (2014). Sedentary behavior and health outcomes among older adults: a systematic review. BMC Public Health.

[10] Harrington DM, Barreira TV, Staiano AE, Katzmarzyk PT (2014). The descriptive epidemiology of sitting among US adults, NHANES 2009/2010. J Sci Med Sport.

[11] Matthews CE, Chen KY, Freedson PS, Buchowski MS, Beech BM, Pate RR (2008). Amount of time spent in sedentary behaviors in the United States, 2003-2004. Am J Epidemiol.

[12] Davis MG, Fox KR, Hillsdon M, Coulson JC, Sharp DJ, Stathi A (2011). Getting out and about in older adults: the nature of daily trips and their association with objectively assessed physical activity. Int J Behav Nutr Phys Act.

[13] Copeland JL, Clarke J, Dogra S (2015). Objectively measured and self-reported sedentary time in older Canadians. Prev Med Rep.

[14] Dogra S, Ashe MC, Biddle SJH, Brown WJ, Buman MP, Chastin S (2017). Sedentary time in older men and women: an international consensus statement and research priorities. Br J Sports Med.

[15] Harvey JA, Chastin SFM, Skelton DA (2013). Prevalence of sedentary behavior in older adults: a systematic review. Int J Environ Res Pub Health.

[16] Atkin AJ, Gorely T, Clemes SA, Yates T, Edwardson C, Brage S (2012). Methods of measurement in epidemiology: sedentary behaviour. Int J Epidemiol.

[17] Aguilar-Farias N, Brown WJ, Peeters GM (2014). ActiGraph GT3X+ cut-points for identifying sedentary behaviour in older adults in free-living environments. J Sci Med Sport.

[18] Gorman E, Hanson HM, Yang PH, Khan KM, Liu-Ambrose T, Ashe MC (2014). Accelerometry analysis of physical activity and sedentary behavior in older adults: a systematic review and data analysis. Eur Rev Aging Phys Act.

[19] Tudor-Locke C, Camhi SM, Troiano RP. A catalog of rules, variables, and definitions applied to accelerometer data in the National Health and Nutrition Examination Survey, 2003–2006. Prev Chronic Dis. 2012;9:E113. 10.5888/pcd9.110332 PMC3457743 22698174

[20] Moher D, Liberati A, Tetzlaff J, Altman DG, Group TP (2009). Preferred reporting items for systematic reviews and meta-analyses: the PRISMA statement. PLoS Med.

[21] Sallis JF, Saelens BE (2000). Assessment of physical activity by self-report: status, limitations, and future directions. Res Q Exerc Sport.

[22] Kelly P, Fitzsimons C, Baker G (2016). Should we reframe how we think about physical activity and sedentary behaviour measurement? Validity and reliability reconsidered. Int J Behav Nutr Phys Act.

[23] Zweig MH, Campbell G (1993). Receiver-operating characteristic (ROC) plots: a fundamental evaluation tool in clinical medicine. Clin Chem.

[24] Bland JM, Altman DG (1986). Statistical methods for assessing agreement between two methods of clinical measurement. Lancet.

[25] Bourke AK, Ihlen EA, Van de Ven P, Nelson J, Helbostad JL (2016). Video analysis validation of a real-time physical activity detection algorithm based on a single waist mounted tri-axial accelerometer sensor. Conf Proc IEEE Eng Med Biol Soc.

[26] Chudyk AM, McAllister MM, Cheung HK, McKay HA, Ashe MC (2017). Are we missing the sitting? Agreement between accelerometer non-wear time validation methods used with older adults’ data. Cogent Med.

[27] Keadle SK, Shiroma EJ, Kamada M, Matthews CE, Harris TB, Lee IM (2017). Reproducibility of accelerometer-assessed physical activity and sedentary time. Am J Prev Med.

[28] Keadle SK, Shiroma EJ, Freedson PS, Lee IM (2014). Impact of accelerometer data processing decisions on the sample size, wear time and physical activity level of a large cohort study. BMC Public Health.

[29] Koster A, Shiroma EJ, Caserotti P, Matthews CE, Chen KY, Glynn NW (2016). Comparison of sedentary estimates between activPAL and hip- and wrist-worn ActiGraph. Med Sci Sports Exerc.

[30] Rosenberg D, Godbole S, Ellis K, Di C, Lacroix A, Natarajan L (2017). Classifiers for accelerometer-measured behaviors in older women. Med Sci Sports Exerc.

[31] Sasaki JE, Hickey AM, Staudenmayer JW, John D, Kent JA, Freedson PS (2016). Performance of activity classification algorithms in free-living older adults. Med Sci Sports Exerc.

[32] Wanner M, Martin BW, Meier F, Probst-Hensch N, Kriemler S (2013). Effects of filter choice in GT3X accelerometer assessments of free-living activity. Med Sci Sports Exerc.

[33] Evenson KR, Wen F, Herring AH, Di C, LaMonte MJ, Tinker LF (2015). Calibrating physical activity intensity for hip-worn accelerometry in women age 60 to 91 years: the Women's Health Initiative OPACH calibration study. Prev Med Rep.

[34] Bai J, Di C, Xiao L, Evenson KR, LaCroix AZ, Crainiceanu CM (2016). An activity index for raw accelerometry data and its comparison with other activity metrics. PLoS One.

[35] Hart TL, Swartz AM, Cashin SE, Strath SJ (2011). How many days of monitoring predict physical activity and sedentary behaviour in older adults. Int J Behav Nutr Phys Act.

[36] Kocherginsky M, Huisingh-Scheetz M, Dale W, Lauderdale DS, Waite L (2017). Measuring physical activity with hip accelerometry among U.S. older adults: How many days are enough?. PLoS One.

[37] Hutto B, Howard VJ, Blair SN, Colabianchi N, Vena JE, Rhodes D (2013). Identifying accelerometer nonwear and wear time in older adults. Int J Behav Nutr Phys Act.

[38] Klenk J, Buchele G, Lindemann U, Kaufmann S, Peter R, Laszlo R (2016). Concurrent validity of activPAL and activPAL3 accelerometers in older adults. J Aging Phys Act.

[39] Wullems JA, Verschueren SMP, Degens H, Morse CI, Onambélé GL (2017). Performance of thigh-mounted triaxial accelerometer algorithms in objective quantification of sedentary behaviour and physical activity in older adults. PLoS One.

[40] Landry GJ, Falck RS, Beets MW, Liu-Ambrose T (2015). Measuring physical activity in older adults: calibrating cut-points for the MotionWatch 8. Front Aging Neurosci.

[41] Choi L, Ward SC, Schnelle JF, Buchowski MS (2012). Assessment of wear/nonwear time classification algorithms for triaxial accelerometer. Med Sci Sports Exerc.

[42] Sirard John R., Forsyth Ann, Oakes J. Michael, Schmitz Kathryn H. (2011). Accelerometer Test-Retest Reliability by Data Processing Algorithms: Results from the Twin Cities Walking Study. Journal of Physical Activity and Health.

[43] Pavey TG, Gomersall SR, Clark BK, Brown WJ (2016). The validity of the GENEActiv wrist-worn accelerometer for measuring adult sedentary time in free living. J Sci Med Sport.

[44] Freedson PS, John D (2013). Comment on “estimating activity and sedentary behavior from an accelerometer on the hip and wrist”. Med Sci Sports Exerc.

[45] Kozey-Keadle S, Libertine A, Lyden K, Staudenmayer J, Freedson PS (2011). Validation of wearable monitors for assessing sedentary behavior. Med Sci Sports Exerc.

[46] Clarke-Cornwell AM, Farragher TM, Cook PA, Granat MH (2016). Empirically derived cut-points for sedentary behaviour: are we sitting differently?. Physiol Meas.

[47] Peterson NE, Sirard JR, Kulbok PA, DeBoer MD, Erickson JM (2015). Validation of accelerometer thresholds and inclinometry for measurement of sedentary behavior in young adult university students. Res Nurs Health.

[48] Johannsen DL, DeLany JP, Frisard MI, Welsch MA, Rowley CK, Fang X (2008). Physical activity in aging: comparison among young, aged, and nonagenarian individuals. J Appl Physiol.

[49] Strath SJ, Pfeiffer KA, Whitt-Glover MC (2012). Accelerometer use with children, older adults, and adults with functional limitations. Med Sci Sports Exerc.

[50] Evenson KR, Terry JW (2009). Assessment of differing definitions of accelerometer nonwear time. Res Q Exerc Sport.

[51] Trost SG, Loprinzi PD, Moore R, Pfeiffer KA (2011). Comparison of accelerometer cut points for predicting activity intensity in youth. Med Sci Sports Exerc.

[52] Freedson P, Bowles HR, Troiano R, Haskell W (2012). Assessment of physical activity using wearable monitors: recommendations for monitor calibration and use in the field. Med Sci Sports Exerc.

[53] Pavey TG, Gilson ND, Gomersall SR, Clark B, Trost SG (2017). Field evaluation of a random forest activity classifier for wrist-worn accelerometer data. J Sci Med Sport.

[54] Lyden K, Keadle SK, Staudenmayer J, Freedson PS (2014). A method to estimate free-living active and sedentary behavior from an accelerometer. Med Sci Sports Exerc.

[55] Rowlands AV, Olds TS, Hillsdon M, Pulsford R, Hurst TL, Eston RG (2014). Assessing sedentary behavior with the GENEActiv: introducing the sedentary sphere. Med Sci Sports Exerc.

[56] Rowlands AV, Yates T, Olds TS, Davies M, Khunti K, Edwardson CL (2016). Sedentary sphere: wrist-worn accelerometer-brand independent posture classification. Med Sci Sports Exerc.

[57] Lyden K, Kozey Keadle SL, Staudenmayer JW, Freedson PS (2012). Validity of two wearable monitors to estimate breaks from sedentary time. Med Sci Sports Exerc.

[58] Lyden K, Keadle SK, Staudenmayer J, Freedson PS (2017). The activPAL™ accurately classifies activity intensity categories in healthy adults. Med Sci Sports Exerc.

[59] Ryde GC, Gilson ND, Suppini A, Brown WJ (2012). Validation of a novel, objective measure of occupational sitting. J Occup Health.

[60] Steeves JA, Bowles HR, McClain JJ, Dodd KW, Brychta RJ, Wang J (2015). Ability of thigh-worn ActiGraph and activPAL monitors to classify posture and motion. Med Sci Sports Exerc.

[61] Choi L, Liu Z, Matthews CE, Buchowski MS (2011). Validation of accelerometer wear and nonwear time classification algorithm. Med Sci Sports Exerc.

[62] Troiano RP, Berrigan D, Dodd KW, Masse LC, Tilert T, McDowell M (2008). Physical activity in the United States measured by accelerometer. Med Sci Sports Exerc.

[63] JOHN DINESH, FREEDSON PATTY (2012). ActiGraph and Actical Physical Activity Monitors. Medicine & Science in Sports & Exercise.

