# Supplementary material for: Validity of objective methods for measuring sedentary behaviour in older adults: a systematic review
Source: Int J Behav Nutr Phys Act. 2018 Nov 26;15:119. doi: 10.1186/s12966-018-0749-2 (PMC6260565; doi:10.1186/s12966-018-0749-2)
Supplement: Supplementary file 1 — Definitions and descriptions of test-retest reliability and validity for assessment of accelerometers and inclinometers. (DOCX 15 kb) [file 12966_2018_749_MOESM1_ESM.docx]

**ADDITIONAL FILE 1. Definitions and descriptions of test-retest reliability and validity for assessment of accelerometers and inclinometers**

***Reliability of accelerometers for measuring sedentary behaviour***

Reliability of accelerometers refers to the consistency in accelerometer readings. One common type of reliability is test-retest reliability, which examines consistency in readings over time. For example, researchers may assess whether an accelerometer classifies the same activities as SB at two or more time points. Reliability testing is often used to determine the number of days of data collection that is needed in order to capture individuals’ usual patterns of SB. This is particularly important to understand in older adults, for whom adherence to 7-day accelerometer protocols is low and for whom increasing age is associated with fewer wear days [1]. Reliability is typically estimated with the intra-class correlation coefficient (ICC) for continuous data [2] like accelerometer data.

Comparisons of accelerometer readings between different filters are also examinations of consistency, and therefore, are treated as examinations of reliability for this paper. For example, researchers may examine whether two filters used with an ActiGraph accelerometer provide similar results.

***Validity of accelerometers for measuring sedentary behaviour***

In this context, validity refers to the extent to which an accelerometer accurately measures SB. Validity may be assessed in laboratory settings or in free-living environments. Two types of validity are of interest in assessing the accuracy of accelerometers: criterion validity and concurrent validity. Criterion validity refers to the extent to which the findings from the measure of interest (accelerometer) agree with the findings produced from using a ‘gold standard’ measure [3]. For assessing the criterion validity of an accelerometer, the gold standard is typically calorimetry or direct observation of behaviour. The second type of validity is concurrent validity, which refers to the extent to which findings from a measure of interest (an accelerometer) agree with the findings produced from another measure of the same phenomena (e.g., another type of accelerometer) [3].

Because accelerometer counts can be analysed for varying epoch lengths, ranging from 1 to 60 seconds, validity is analysed for specific epochs. To assess validity requires either (a) using an a-priori cut-point between SB and non-SB behaviours or (b) assessing a range of cut-points. To assess a range of cut-points, researchers typically evaluate which ones optimise sensitivity (% of SB correctly classified as SB) and specificity (% of non-SB correctly classified as non-SB). The area under the receiver operating characteristics (ROC) curve is often reported along with sensitivity and specificity. Values closer to 1.00 indicate more accurate classification of SB, and values closer to 0.5 indicate less accurate classification of SB [4]. However, these methods are not always used for assessing the validity of SB as there is no consensus in the literature about the most appropriate methods for evaluating validity [3]. Statistical models (e.g., non-parametric or regression models) or Bland–Altman methods [5] may be used in addition to, or alternatively to, ROC methods, to examine relationships or agreement between the accelerometer of interest and the criterion or concurrent measure.

***Considerations in assessing the reliability and validity of accelerometer data collected in free-living conditions***

For monitors that participants can remove easily, other influences on reliability and validity estimates of SB are the number of hours per day and days of the observation period that a monitor must be worn for data to be included in analysis. Most studies that used ActiGraph accelerometers with adults have used the rules that the monitor must be worn ≥10 hours per day and for ≥4 days [6]. Another consideration is the definition of ‘non-wear’ time, the number of consecutive minutes that a monitor captures no movement (e.g., zero cpm). The non-wear algorithm selected for processing data affects estimates of SB [6] because a long string of zeros could represent either time that the monitor was not worn or an extended time period in which the monitor wearer is still. Most studies of adults have defined non-wear time as ≥60 minutes of zero cpm, with some studies allowing for brief interruptions [6]. The Troiano algorithm [7] uses this definition of non-wear time with allowances of 1-2 minutes of <100 cpm. The Choi algorithm [8] defines non-wear time as ≥90 minutes of consecutive zero cpm with an allowance of 2 minutes of non-zero cpm if the 30 minutes before or after the interruption consists of consecutive zeros. These algorithms were developed for use with uniaxial accelerometers but have been used with triaxial accelerometers [8].

References

1. Kocherginsky M, Huisingh-Scheetz M, Dale W, Lauderdale DS, Waite L. Measuring physical activity with hip accelerometry among U.S. older adults: How many days are enough? PLoS ONE 2017;12.

2. Sallis JF, Saelens BE. Assessment of physical activity by self-report: status, limitations, and future directions. Res Q Exerc Sport. 2000, 71:S1-14.

3. Kelly P, Fitzsimons C, Baker G. Should we reframe how we think about physical activity and sedentary behaviour measurement? Validity and reliability reconsidered. Int J Behav Nutr Phys Act. 2016;13:32.

4. Zweig MH, Campbell G. Receiver-operating characteristic (ROC) plots: a fundamental evaluation tool in clinical medicine. Clin Chem. 1993;39:561-77.

5. Bland JM, Altman DG. Statistical methods for assessing agreement between two methods of clinical measurement. Lancet. 1986;1:307-10.

6. Tudor-Locke C, Camhi SM, Troiano RP. A catalog of rules, variables, and definitions applied to accelerometer data in the National Health and Nutrition Examination Survey, 2003–2006. Prev Chron Dis. 2012;9:E113.

7. Troiano RP, Berrigan D, Dodd KW, Masse LC, Tilert T, McDowell M. Physical activity in the United States measured by accelerometer. Med Sci Sports Exerc. 2008;40:181-8.

8. Choi L, Ward SC, Schnelle JF, Buchowski MS. Assessment of wear/nonwear time classification algorithms for triaxial accelerometer. Med Sci Sports Exerc. 201244:2009-16.
